# Endoscopic dacryocystorhinostomy to treat congenital nasolacrimal canal dysplasia: a retrospective analysis in 40 children

**DOI:** 10.1186/s12886-019-1256-1

**Published:** 2019-12-03

**Authors:** Yan-Hui Cui, Cheng-Yue Zhang, Wen Liu, Qian Wu, Gang Yu, Li Li, Wen-Bin Wei

**Affiliations:** 1 Department of Ophthalmology, Beijing Children’s Hospital, National Center for Children’s Health, National Key Discipline of Pediatrics, Capital Medical University, Ministry of Education, Beijing, 100045 China; 2 Beijing Tongren Eye Center, Beijing Tongren Hospital, Beijing key Laboratory of Intraocular Tumor Diagnosis and Treatment, Beijing Ophthalmology and Visual Science Key Lab, Capital Medical University, 1 Dong Jiao Min Xiang, Dong Cheng District, Beijing, 100730 China

**Keywords:** Nasal endoscopy, Endoscopic dacryocystorhinostomy (EN-DCR), Children, Congenital nasolacrimal duct obstruction (CNLDO), Nasolacrimal canal dysplasia

## Abstract

**Background:**

To investigate the therapeutic effectiveness and safety of endoscopic dacryocystorhinostomy (EN-DCR) to treat congenital nasolacrimal canal dysplasia (CNCD).

**Methods:**

Forty children (50 eyes) with congenital nasolacrimal duct obstruction (CNLDO) and lacrimal bony dysplasia, including 8 children with bony atresia (10 eyes) and 32 with bony stenosis (40 eyes), were recruited in this retrospective study. Standardized EN-DCR was performed in all cases. The postoperative observations included relief of symptoms, fluorescein dye disappearance test (FDDT), syringing of lacrimal passages and anastomotic patency under nasal endoscopy. Patients were followed up for 8–18 months.

**Results:**

Standardized EN-DCR surgery had a success (cure and improvement) rate of 100%, including a cure rate of 82% and an improvement rate of 18%. The cure rate among 40 cases of bony nasolacrimal duct stenosis was 82.5%, while that of 10 cases of bony nasolacrimal duct atresia was 80%. Statistical analysis showed that nether the receipt of other treatments before surgery nor the type of bony nasolacrimal duct dysplasia affected the cure rate. No significant complications were observed during postoperative follow-up except for four cases (4 eyes) that suffered middle turbinate and nasal mucosal adhesion and two cases with sinusitis.

**Conclusions:**

CNCD is a type of CNLDO that does not respond to conservative and conventional treatment. EN-DCR represents a safe and effective treatment for children with CNCD. In addition, the combination of EN-DCR with lacrimal CT scanning provides advantages over traditional lacrimal surgery in that it has a high success rate with a low incidence of complications.

## Background

Congenital nasolacrimal canal dysplasia (CNCD) is a subtype of common congenital nasolacrimal duct obstruction (CNLDO) [1, 2]. This type of congenital lacrimal duct deformity results from a rudimentary or immature bony nasolacrimal duct. Several studies have used computed tomography (CT) scans to characterize the abnormal features of CNCD [1–5].

Conservative and conventional therapy, such as spontaneous relief, massage, irrigation, lacrimal duct probing, and intubation surgery, are ineffective in CNCD. In recent years, nasal endoscopic surgical techniques have become more frequently used by ophthalmologists and ear-nose-throat physicians to treat lacrimal duct obstruction diseases and dacryocystitis. At present, endoscopic dacryocystorhinostomy (EN-DCR) is generally viewed as the only effective treatment for CNCD [6–8]. However, few reports have described the use of EN-DCR to treat CNCD in children [9–11]. In the present study, we report the clinical data of 40 pediatric cases (50 eyes) of CNCD treated with EN-DCR in our hospital from 2012 to 2016.

## Methods

### Patients

Forty children (50 eyes) with CNCD who underwent EN-DCR in the Department of Ophthalmology, Beijing Children’s Hospital, Capital Medical University, from February 2012 to March 2016 were recruited into this study.

**Fig. 1:**
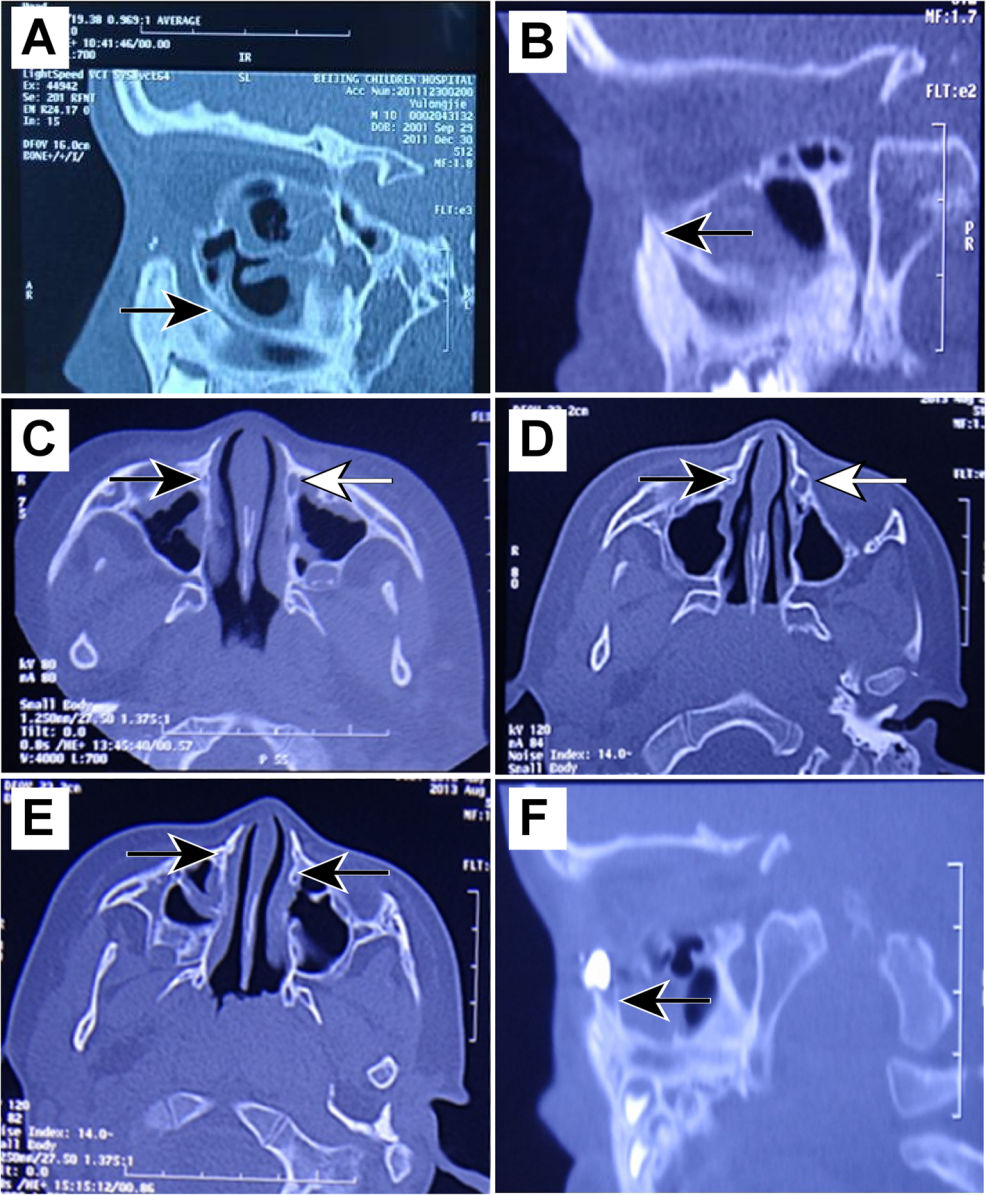
Computed tomography angiography of the lacrimal duct allows congenital nasolacrimal canal dysplasia to be clearly distinguished. (**a, b**) The sagittal position of bony nasolacrimal duct stenosis (black arrow). (**c, d**) The horizontal position of bony nasolacrimal duct stenosis (black arrow) and the contralateral contrast (white arrow). (**e, f**) Both the horizontal and sagittal position show bilateral hypomere bony nasolacrimal duct atresia (black arrow)

The diagnosis of CNCD was based on the history provided by the parents, the presence of symptoms of postpartum tear discharge with eye discharge, tests of the patency of the nasolacrimal canal conducted by irrigating the lacrimal sac, and a plain CT scan examination of the lacrimal duct. All cases completed a lacrimal CT scan within one month before surgery. Based on the report by Yu et al. [1] and Zhang et al. [2], lacrimal bony dysplasia was divided into bone nasolacrimal duct stenosis and bone atresia using CT scans (Fig. 1).

The demographic and clinical information obtained in the patients is summarized in Table 1. The inclusion criteria were CNLDO with lacrimal bony dysplasia and an age of 2–18 years. Exclusion criteria included anomalies of the initial segment of the lacrimal passage, such as an absent inferior lacrimal puncta, absence or atresia of the inferior canal, traumatic lacrimal duct obstruction, lacrimal fractures induced by trauma, or lacrimal sac damage or displacement [12].

**Table 1:** The general clinical and demographic information of subjects

Item	Data of all patients (*n* = 40)
Gender	
Male	26/40 (65%)
Female	14/40 (35%)
Age (years)	
Average	5.5
Median	6
Range	2–14
Side (cases)	
Bilateral	12/40 (30%)
Right	12/40 (30%)
Left	16/40 (40%)
Preoperative treatment (eyes)	
Probing	16/50 (32%)
Intubation	6/50 (12%)
Massaging	28/50 (56%)
Type of bony dysplasia (eyes)	
Stenosis	40/50 (80%)
Atresia	10/50 (20%)
Effects	
Success rate	50/50 (100%)
Cure rate	41/50 (82%)
Improvement	9/50 (18%)
Complications (cases)	
Middle turbinate and nasal mucosal adhesion	4
Nasosinusitis	2
Other	0

The indications for EN-DCR included cases that had been clearly diagnosed as CNCD. This type of case was also suitable for external dacryocystorhinostomy (DCR). In all cases, the parents in this group chose EN-DCR.

### Ethics statement

This study was a retrospective study conducted in accordance with the principles of the Helsinki Declaration and approved by the Institutional Review Committee of Beijing Children’s Hospital. Written informed consent was obtained from the parents of all patients before surgical treatment.

### Surgical procedures

All procedures were performed under general anesthesia. Patients were required to remain supine with the head tilted back 15°. The nasal cavity on the surgery side was shrunk by using 1:10000 adrenaline cotton pieces. An endoscopic surgical system (Karl Storz SE and Co. KG, Tuttlingen, German) and 4.0-mm, 0° rigid nasal endoscopy (XiON GmbH, Berlin, German) were utilized. Other surgical equipment included an ENT power system (Medtronic PLC, Dublin, Ireland), a 4.0-mm ear burr, ophthalmic devices (including 3.0- and 2.0-mm rongeurs), and a Blasky child’s mucosal biting clamp.

**Fig. 2:**
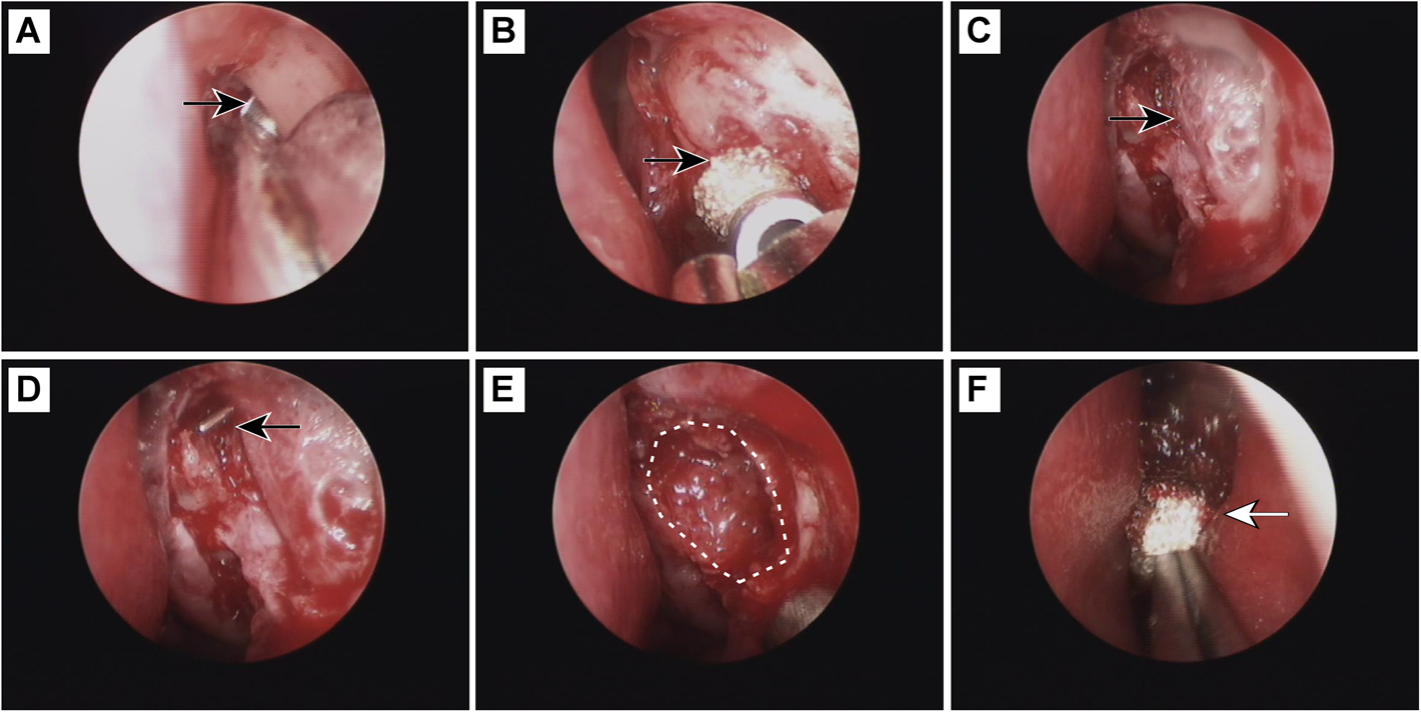
Illustration of the steps of the operation. (**a**) Remove the frontal process of the maxilla and move the sutura lacrimal maxillaris forward with the rongeur (black arrow). (**b**) Use an electric drill to grind the thicker parts of the maxilla frontal process upper bone, if needed (black arrow). (**c**) After producing the bone window, expose the inner wall of the lacrimal sac (black arrow). (**d**) From the lacrimal point, insert the lacrimal probe into the lacrimal sac (black arrow) after endoscopy has verified accurate exposure of the lacrimal sac (black arrow). (**e**) A longitudinal incision is made along the lacrimal sac wall, and a crosscut incision is then made at the top and bottom of the initial incision to form a base at the edge of the lacrimal sac wall and form the mucosal flap. Flip the mucosal flap backward to expose the front mucous membrane of the uncinate process, leaving the lacrimal sac cavity completely open (white outline). (**f**) Blood should be absorbed by stuffing gauze into the lacrimal sac (white arrow), and the lacrimal sac mucosal flap should be pinned to reduce movement and bleeding

The EN-DCR procedure was divided into three steps according to the guidelines for rigid endoscopy [13] (Fig. 2). In step one, a 2.0 × 1.5 cm mucoperiosteal flap was made in the lateral nasal wall using a sickle knife starting from the front of the uncinate process (located mainly above the middle turbinate axillary) and reaching the periosteum. In step two, a bone window was made. As shown in Fig. 1a-c, the frontal process of the maxilla was engaged using a rongeur from the suturae lacrimal maxillaris; the front lacrimal bone was separated and clamped, and a bone window was then formed with a diameter of about l × 1.5 cm. If the upper frontal process of the maxilla bone was thick, it could be ground down with an electric drill. Then, the medial lacrimal sac wall (with a light pale blue color) was exposed. After the lacrimal sac had been exposed, a Bowman probe was placed through the canaliculus into the sac. A vertical incision was then made with a sickle knife, and the medial wall of the sac was removed or the mucosal flap of the lacrimal sac was placed posteriorly, as shown in Fig. 1d-f. In step three, the lacrimal sac was filled with vampire gauze or a gelatin sponge, which was pressed against the lacrimal sac mucosal flap to reduce movement and promote epithelialization of the anastomosis and to attenuate early postoperative bleeding.

### Postoperative treatment and follow up

Systemic antibiotics and hemostasis were administered for three days after surgery. Saline nasal sprays were recommended for use in the month after surgery. A nasal wash was performed beginning on the fourth day using a nose washing apparatus. The first postoperative follow-up was arranged for the seventh day after surgery, when patients were subjected to a lacrimal passage flush. A solution containing tobramycin and dexamethasone (diluted 1:10) was used to flush the lacrimal passage from the upper and the lower punctum. In general, the first flush met with significant resistance and was accompanied by pus discharge and regurgitation. Sustained pressure washing was recommended until flushing became smooth, swallowing was confirmed, and regurgitation disappeared or until the discharge reflux fluid became clear. In the first month after surgery, a lacrimal passage flush was performed weekly; then, it was performed every two weeks for another two months. After one month, anastomotic exploration was routinely performed in all cases under local anesthesia according to the patient’s age, the degree of anastomotic epithelialization, and the timely discovery and cleaning of proliferated granulation tissue around the pore. At 8–18 months after the surgery, anastomotic exploration was again performed. Outpatient follow-up studies included a medical history, a fluorescein dye disappearance test (FDDT), lacrimal passage flush, and anastomotic exploration under nasal endoscopy in children > 6 years old. The evaluation of duct drainage function by FDDT was considered a major endpoint (FDDT 0–1: none or a thin fluorescing marginal tear strip that persists in the conjunctival sac and normal lacrimal drainage function; FDDT 2–3: fluorescein persists in the conjunctival sac and the lacrimal drainage system is obstructed) [13].

### Clinical criteria for surgery outcomes

Surgical effects were divided into cure, improvement, and invalid, according to clinical criteria. Cure was defined as lacrimal sac pore formation in the lateral nasal wall in front of the middle concha, epithelialization under endoscopic observation, no tearing or pus, FDDT = 0, and smooth flush. Improvement was defined as lacrimal sac pore formation in the lateral nasal wall in front of the middle concha, epithelialization under endoscopic observation, symptom relief, FDDT = 1, and flush unobstructed or pressure flush unobstructed. Invalid was defined as no relief of symptoms, FDDT = 2–3, flush unsmooth or pressurized, and pore atresia. Cure and improvement were considered successful surgery, and surgery efficiency was calculated as the sum of the cure rate and the improvement rate [14].

### Statistical analysis

Statistical Package for the Social Sciences (SPSS) software (SPSS Inc. Released 2008. SPSS Statistics for Windows, Version 17.0. Chicago: SPSS Inc.) was used for statistical analysis. The efficacy of preoperative treatment and the effect of type of bony nasolacrimal duct dysplasia on the cure rate were analyzed using the chi-square test, with *P* < 0.05 representing statistical significance.

## Results

Fifty endoscopic DCRs were performed on 40 patients (50 eyes) with CNCD. There were no intraoperative complications. The mean age at surgery was 5.5 years (range 2–14 years). The male to female ratio was 1.9:1 (26:14). Previous interventions included probing in 32% of patients (16/50), massage in 56% (28/50) and intubation in 12% (6/50). The follow-up period was 8–18 months (average 16.5 months). Preoperative plain CT scan of the lacrimal passage revealed 8 bony atresia cases in 20% (10/50) and 32 bony stenosis cases in 80% (40/50). The demographic information and clinical outcomes of cases are summarized in Table 1.

According to the criteria, the success rate was 100%(50/50), the cure rate was 82%(41/50) and the improvement rate was 18%(9/50). Patients in both the nasolacrimal canal stenosis and atresia groups achieved a high effective rate (Table 2). Chi-square analysis showed that the type of nasolacrimal duct dysplasia had no impact on the efficacy of surgical treatment based on the similar cure and improvement rates in both groups (χ^2^ = 0.08, *P* = 0.78). According to Table 3, the chi-square test showed that the cure rate was not affected by treatments performed before EN-DCR (χ^2^ = 1.17, *P* = 0.28).

**Table 2:** The effect of type of bony nasolacrimal duct dysplasia on cure rate

Type	Cure (%)	Improvement (%)	Invalid (%)	Total
St.	33 (82.5)	7 (17.5)	0	40
At.	8 (80)	2 (20)	0	10
Total	41 (82)	9 (18)	0	50

St. = Stenosis of the nasolacrimal duct; At. = Atresia of the nasolacrimal duct

The chi-square test showed that the cure rate was not affected by type of bony nasolacrimal duct dysplasia (χ^2^ = 0.08, *P* = 0.78, no statistically significant difference)

**Table 3:** The effect of preoperative treatment on cure rate

Group	Cure (%)	Improvement (%)	Invalid (%)	Total
T	20 (91)	2 (9)	0	22
N	21 (75)	7 (25)	0	28
Total	41	9	0	50

T = treated before surgery, such as receiving intubation and probing

*N* = not treated before surgery

The chi-square test showed that the cure rate was not affected by other treatments before endoscopic dacryocystorhinostomy (χ^2^ = 1.17, *P* = 0.28, no statistically significant difference)

**Fig. 3:**
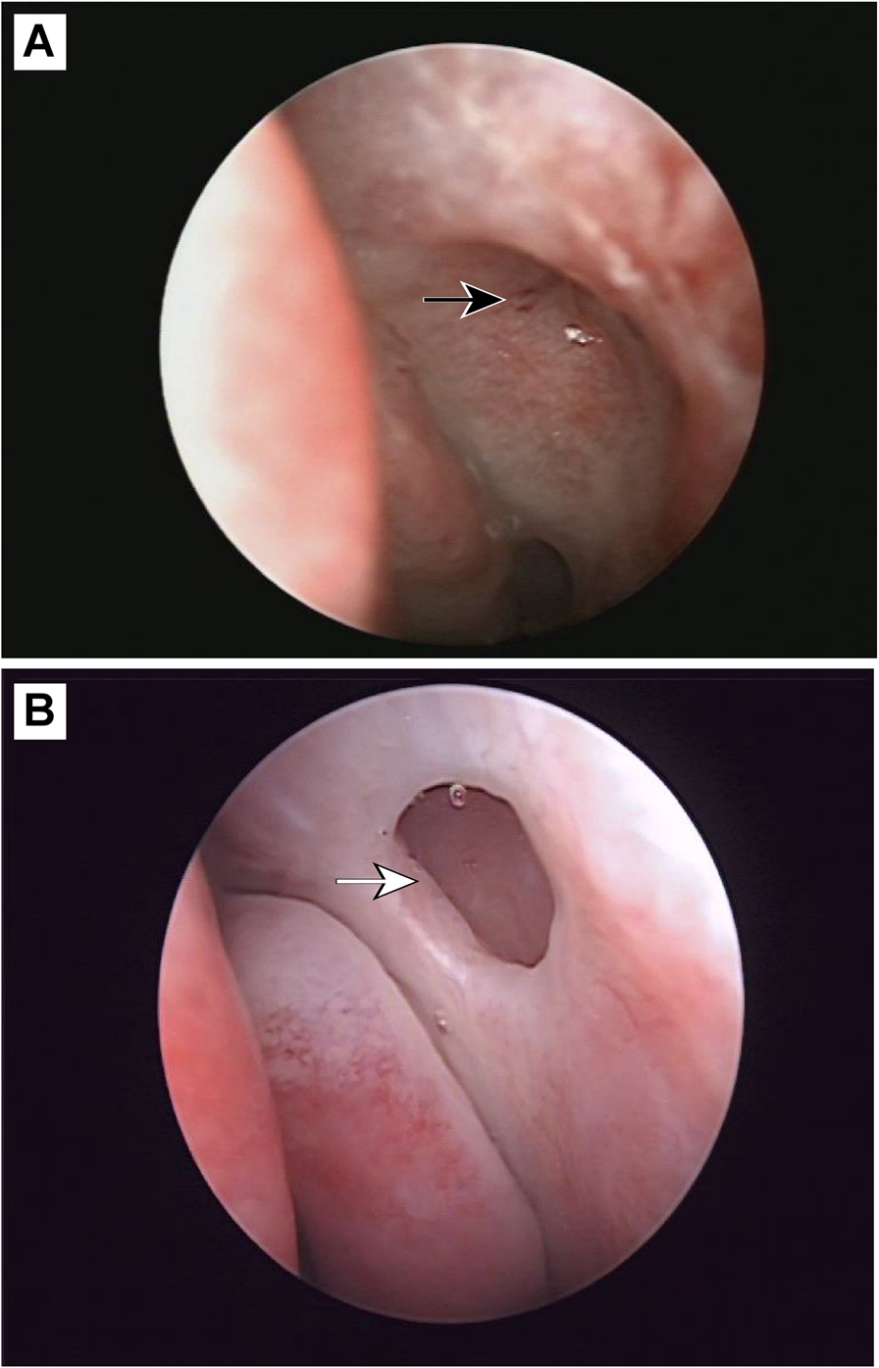
Postoperative anastomotic opening. (**a**) The anastomotic opening is healing but still shows mild edema at 1 month postoperative (black arrow). (**b**) The anastomotic opening at 12 months postoperative, with epithelization (white arrow)

We explored the stoma one month after surgery in patients who received general or local anesthesia. Among the 40 patients, 6 cases (8 eyes) had no follow up on time, and the other 34 cases (42 eyes) were followed up on time. These had round or oval nasal anastomosis with healed mucosa with a maximum diameter of 6–7 mm (Fig. 3). However, visible mucosal thickening and edema remained, with bleeding upon tactile investigation. Among these 42 eyes, 32 had granulation in varying degrees. The early postoperative granulated tissue around the stoma was vulnerable and bled easier, making it accessible for removal using nasal mucosa pliers. After clearing the granulation tissue, bleeding was stopped with a gelatin sponge. Six cases (7 eyes) were followed up later than one month (2–3 months), by which time the mucosal edema was not obvious and the stoma had basically epithelialized. Four eyes had visible granulation tissue, which was surgically removed. Twenty-eight cases (36 eyes) who received anastomotic probing under general or local anesthesia were followed up after 8–18 months. The probing results showed that the anastomotic hole was round or oval and showed no atresia. The diameter of the anastomotic hole was approximately 2–5 mm, and the maximum pore diameter was 5 mm (as shown in Fig. 3). The mucosa around the anastomosis was highly epithelialized and smooth without granulation tissue hyperplasia.

Few patients had significant complications during the operation or postoperative follow-up period. Adhesion of the middle turbinate to the lateral wall nasal mucosa was found in four cases (4 eyes) and separated through exploratory surgery. Four patients complained of occasional headache, and two were diagnosed with nasosinusitis. Further observation was needed in these cases because no nasosinusitis was found in these four patients prior to surgery.

## Discussion

CNCD is a particular type of CNLDO. The vast majority of cases of CNCD are initially diagnosed as common CNLDO and treated conservatively or conventionally, such as with massage, lacrimal duct probing, and intubation surgery. When these treatments prove ineffective, a CT scan examination of the lacrimal duct may reveal CNCD. A CT scan can precisely delineate the shape, direction, and abnormal structure of the bony nasolacrimal duct and provide a clear anatomical characterization of the abnormal nasolacrimal canal. These data can be used to categorize abnormalities of the nasolacrimal canal as significant stenosis or even atresia [2]. Prior to the use of lacrimal CT scans, many children suffered repeated lacrimal probing, which can cause complications such as false passages and punctal tears [12]. A CT scan can help diagnose congenital nasolacrimal duct abnormalities, thereby avoiding lacrimal tissue damage. Moreover, a CT scan of the lacrimal passage can not only accurately determine the location and degree of obstruction but also help to determine the developmental level of the agger nasi and its anatomic relationship with the lacrimal sac. In addition, it can help to identify abnormalities in the paranasal sinuses and lacrimal sac and can provide especially valuable information in patients with surgical histories. We previously performed CT scans in children with failed lacrimal probing and found that these children had bony nasolacrimal canal stenosis or atresia [1, 2]. We recommend that these children should stop receiving lacrimal probing and should undergo EN-DCR.

EN-DCR is now a widely-accepted technique for managing nasolacrimal duct obstructions with success rates comparable to those of external DCR [15–19]. EN-DCR represents a mature technology used to treat complex nasal lacrimal duct obstructions in children [12]. Previous studies have achieved success rates in pediatric EN-DCR of 58–100% [20]. Leibovitch et al. [21] reported that while the anatomical patency rate was 100% (endoscopic anastomotic opening), the success rate (complete disappearance of symptoms) was only 92%. In our study of the application of EN-DCR in the treatment of CNCD, the success rate was 100% (50/50), the cure rate was 82% (41/50), and the improvement rate was 18% (9/50), similar to the results obtained in previous studies. In particular, combining EN-DCR with lacrimal CT scan provides advantages over traditional lacrimal surgery. In addition, few patients experienced significant complications during either the operation or the postoperative follow-up period. Adhesion of the middle turbinate to the lateral wall nasal mucosa was found in four cases (4 eyes) and was separated through exploratory surgery. In our experience, EN-DCR is not recommended in children younger than two years. In cases in which bone dysplasia is observed on CT scan, age is not a key consideration for EN-DCR. These children also have serious dacryocystitis symptoms, and their intubation success rate is very low. Additionally, repeated intubation or probing can easily cause false passage and infection. If EN-DCR must be performed in very young children, the skill of the surgeons and the equipment used must be optimal.

For children with lacrimal duct obstruction, the initial EN-DCR can not only avoid skin scarring but also protect the exhaust pump function of the orbicularis muscle and the medial canthal ligament. Because it involves the use of sophisticated endoscopic equipment and intranasal devices, EN-DCR may obtain a clearer operating field, cause fewer injuries, and result in less bleeding and shorter operation times than are achieved using external DCR. These advantages are important for children who need general anesthesia. Postoperatively, external operations require nasal packing, daily flush washing early in the postoperative stages, wound care, and stitches, and they therefore have a higher chance of infection. While transnasal surgery requires simpler postoperative treatment, it is also necessary to perform intranasal anastomotic exploration in young children during the early postoperative stage (within one month), and this is usually performed under general anesthesia. We explored the stoma using sevoflurane under general anesthesia and treated tissues that exhibited granulation in young children.

Because of the small anatomical space in young children, a 2.7-mm endoscope [22] as well as a nasal trumpet are recommended. However, some scholars have held that a 4.0-mm endoscope can be used in children younger than one year because it can provide better lighting and a wider viewing angle; however, the results depend on the endoscopic surgeon’s skill level, personal preferences, and device conditions [6]. In this study, the youngest child was 2 years old, and a 4.0-mm endoscope was successfully used in this patient, providing better lighting and a wider visual field than was achievable with a 2.7-mm endoscope. The medial wall of the lacrimal sac may become thick due to inflammation, and we therefore recommend using a corneal puncture with a 15° blade; however, some surgeons may be more accustomed to using a sharp sickle knife, a myringotome, a high-frequency electric knife, or a laser [22]. Experts agree that achieving successful outcomes in pediatric DCRs requires adequately sized and positioned osteotomy, full-length sac marsupialization, and a 360° mucosa-to-mucosa approximation to facilitate healing [23].

A large bone window is recommended when exposing the inner lacrimal wall as this will improve the opening rate of the pore [21]. However, a small bone window will be susceptible to blockage by a postoperative blood scab, exudate, or granulation tissue, resulting in a failed operation.

The use of lacrimal stents in pediatric cases is controversial [24–26]. Some studies have recommended that a lacrimal silicone tube should be placed in all EN-DCR patients for four weeks to expand the tear duct and sac opening instead of allowing the lacrimal sac to remain open [23]. We conclude that in cases with bony nasolacrimal duct stenosis or atresia, the lacrimal sac and the surrounding bones should be allowed to develop normally or even expand slightly so that the lacrimal bone hole fits easily with the nasal mucosal flap and nasal mucosa. If there is no canalicular lesion, it is not necessary to place any anastomotic support. This approach also avoids a second surgery to remove the tube. Ensuring a good nasal mucosal flap and lacrimal sac is important for the success of the operation. Vampire gauze or gelatin sponge can also be used as a short-term support for the anastomosis of the lacrimal sac and to press the nasal mucosa and lacrimal mucosa together to stop bleeding. By the time the vampire gauze or gelatin sponge is absorbed, anastomotic mucosal healing should be largely complete. Therefore, the decision to place a lacrimal duct support depends on the child’s condition. An important prerequisite to ensuring the success of this surgery is a superb endoscopic sinus surgery technique and a deep familiarity with the sinonasal anatomy and the structural relationships around the eyes. In this study, none of the cases underwent lacrimal duct dilatation or catheter tube placement.

### Limitations

The limitations of the current study include its retrospective and single-center design. The limited sample size prevented group comparisons.

## Conclusions

In summary, CNCD is a type of CNLDO that does not respond to conservative and conventional treatment. EN-DCR is a safe and effective treatment for children with CNCD. In addition, the combination of EN-DCR with lacrimal CT scan provides advantages over traditional lacrimal surgery: it has a high success rate and a low incidence of complications. Multicenter studies should be performed to confirm these conclusions.

## Abbreviations

CNCD: Congenital nasolacrimal canal dysplasia
CNLDO: Congenital nasolacrimal duct obstruction
CT: Computed tomography
DCR: Dacryocystorhinostomy
EN-DCR: Endoscopic dacryocystorhinostomy
FDDT: Fluorescein dye disappearance test
SPSS: Statistical Package for the Social Sciences
